# Managing AI is managing complexity - An interview with Rahul C. Basole

**DOI:** 10.1007/s12525-022-00585-5

**Published:** 2022-09-01

**Authors:** Rainer Alt

**Affiliations:** Information Systems Institute, Leipzig University, Grimmaische Str. 12, 04109 Leipzig, Germany

## Background information

This interview with Rahul C. Basole has emerged from his current role as member of Electronic Markets’ Advisory Board, which he has assumed in 2020. Rahul is Managing Director and Global Lead for Visualization and Interaction Science (VIS) at Accenture Applied Intelligence (Accenture AI). As a global professional services company, Accenture combines strategy and consulting, interactive, technology and operations services in the fields cloud, digital and security. Its 710,000 people serve clients in more than 120 countries and more than 40 industries worldwide. Applied Intelligence is Accenture’s approach to scaling artificial intelligence (AI) and machine learning (ML) for clients by embedding AI-powered data, analytics, and automation capabilities into business workflows. The VIS capability includes a global group of visualization experts, data scientists, information designers, and enterprise architects that work in application areas such as human-centered design, interactive data experiences, immersive visual analytics, digital twins, and enterprise simulators. In his prior role, Rahul was an Associate Professor in the College of Computing at the Georgia Institute of Technology and visiting faculty at Stanford University. In his research, he has authored influential publications on digital ecosystems (de Reuver et al. 2018), the complexity of service value networks (Basole and Rouse 2008), and on ecosystem visualization (Basole 2009). He has been named to Stanford’s 2021 Global List of Top 2% Scientists and holds a Master of Science degree in Industrial and Operations Engineering from the University of Michigan as well as a PhD in Industrial and Systems Engineering from the Georgia Institute of Technology.

**Figure:**
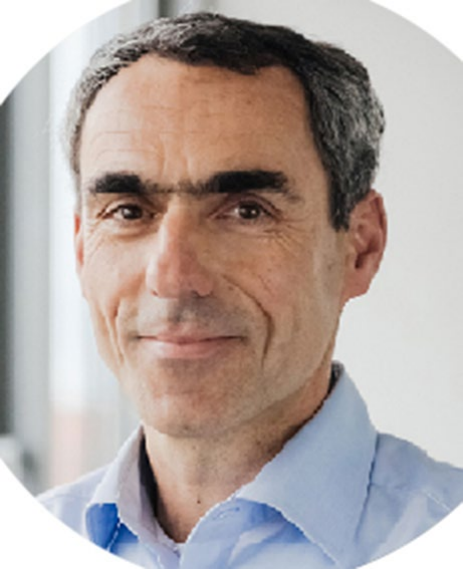
Rahul C. Basole

**Figure:**
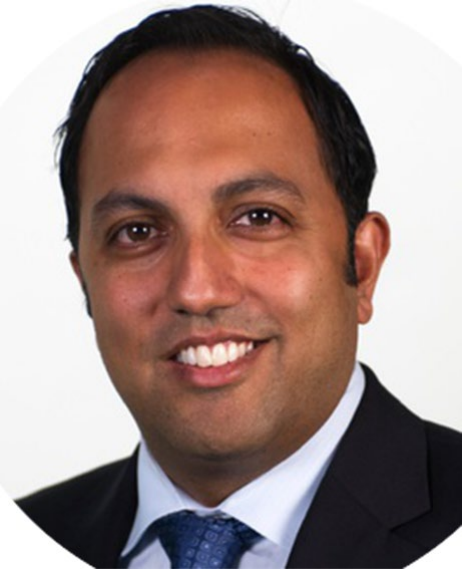
Rainer Alt

## What are the main topics in your AI practice?

Although we know that the need to digitize and to become data-driven has been a priority for companies for decades, the past years have seen a significant momentum shift. Since assuming my current position at Accenture AI three years ago, this shift in the digital transformation of enterprises may be observed across all sizes, functions, industries, and geographies. It has been particularly pronounced over the past 24 months, during which major transformation initiatives were expedited from multiple years to, in some instances, just a few months. We know that the particularly disruptive impact of Covid-19 on business and society accelerated data-led transformations. Executives were required to rethink their digital strategies more fundamentally along several dimensions, both internally and externally. It not only elevated the impetus of moving data and applications to the cloud, but also the need to unlock trapped or entirely new value streams through analytics, automation, and AI. All of this needed to happen at speed and at scale, beyond the proof of concept or pilot stage, where many of these initiatives often failed in the past. To address this, Accenture pursues a one team approach that brings together strategy and domain experts, data scientists and engineers, visualization and interaction scientists, human-centered designers, advanced AI/ML engineers, solution architects, and talent and organization specialists, just to name a few key roles. Besides our own network, we strongly co-create value with our clients and our extensive partner ecosystem. What all of this should highlight is that data, analytics, and AI initiatives are not trivial endeavours. They require a holistic approach, a breadth and depth of capabilities, and an orchestration of a wide range of stakeholders.

## What is the role of organizational transformation?

Both practice and research have shown that digital enablement of people, processes, organizations, and ecosystems is essential to the growth and survival of firms. Many of the traditional constraints that we know from information systems research, such as change and implementation of business processes and organizational culture as well as values, are still there. In fact, the biggest challenge of becoming data-driven may not have to do with technology at all. What has changed is the realization that change is inevitable, that businesses must face it and learn to manage it. Today, the pressure to accelerate digital transformation not only originates from the technological side, but rather from every angle, be it organizational, political, or cultural in nature. Many of our clients are establishing specific functions for digital transformation and they are realizing that besides the actual technological solutions the entire organization requires a new mindset. This comprises the ability to adapt to continuous change, and the leadership to overcome established processes and organizational structures, which are often hardened over several decades. To become data-driven, these must be revisited, redesigned, or even replaced with entirely new ones, and that change is challenging. Although a “one size approach” will not fit all, we observe patterns across industries that companies with a dedicated data and analytics hub and change management capabilities as well as learning, upskilling, and community engagement programs, are likely to succeed in their AI endeavors.

## What are the technological challenges?

Today, enterprises are creating and consuming a vast amount of data from inside and outside their organization. Data can come from many different sources, including existing information systems, through APIs to suppliers and partners, from IoT devices providing streaming and right-time data, or as digital signals and traces from the broader business ecosystem. Making this data available is key and Accenture emphasizes this with its cloud-first strategy. Digitalization unleashes its real power when the business becomes cloud-enabled and moves all this data into a place that is available to the entire enterprise. It is the basis for a fully instrumented enterprise that collects all signals in right time and enables decision-makers to execute on them. The goal is to create a “single view of X” where you may fill in X with different aspects of the enterprise, for example, the single view of customer, the single view of supply chain, or the single view of product. It is important since there is a wealth of data about almost any entity of interest and bringing it together into a single view ultimately creates the foundation for analytics, automation, and AI. If we consider that cloud is the enabler, data is the driver, and AI is the differentiator, I would then highlight three challenges. First, it requires selecting a cloud strategy and building an appropriate technology stack around it. Second, a multi-layer data environment is necessary where data is not just captured and integrated with high data quality and governance, but also progressively enriched and linked to other data with semantics, and ultimately made available for activation, reporting, exploration, discovery, and self-service across the enterprise. Having data in the cloud and hyper-integrated then allows the creation of the single view of X. Third, organizations need to architect an integrated, high-performance system, where data is available at the right time and at the right granularity and shared across all functions on the analytics layers to produce coordinated insights, decisions, and actions, with a feedback loop to the data layer that enables continuous learning.

## What is the role of digital platforms?

**Fig. 1:**
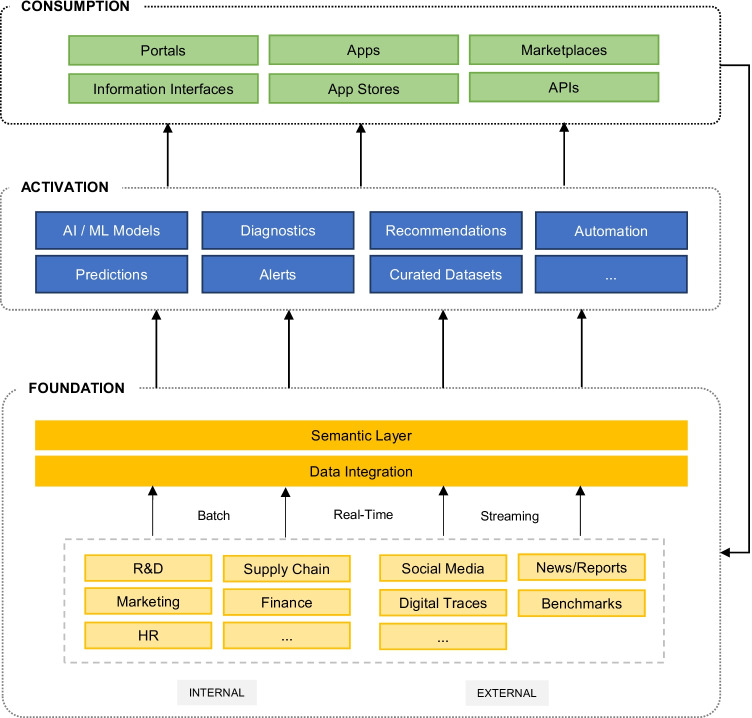
Simplified model of an enterprise (data, analytics, and AI) technology stack (Source: Accenture AI)

Platform-centric thinking is essential to the growth and success of digital firms today. Platforms enable firms to scale their offerings, connect with their ecosystem, and create new innovative products and services. The intent is to reduce the complexity by moving from a point-to-point exchange of data towards an orchestrated space. Contrary to existing beliefs, there is no single digital platform that powers the needs of digital organizations. Instead, what we are observing is that successful data-led organizations will orchestrate a complex set of platforms, at different levels, each responsible for a particular aspect of the digital enterprise. Broadly considered, I see three levels at which digital platforms operate (see Figure 1). At the foundation of it all is a robust, integrated, and scalable data infrastructure layer. Digital platforms associated with this layer enable that data is captured, curated, organized, progressively enriched, and governed. Really exciting is what happens on top of this foundational data layer. The central promise of the activation layer is to convert the potential value of data into realized value. At this layer, data scientists and AI engineers leverage digital platforms to build, test, and deploy models, create alerts, make predictions, and generate recommendations. Finally, we have a consumption layer. At this layer, data, analytics, and AI are consumed by the end-user through a variety of platforms. Applications and services are made available and interacted with through marketplaces, portals, app stores, exchanges, and interactive interfaces.

## Can you elaborate on the AI functionalities?

Analytics and AI are exciting since we can operate with much larger data sets that are integrated across different areas today. This allows us to build more advanced, more accurate, and more targeted ML models about entities of interest that can act in real-time. AI functionalities are present across the entire applied intelligence pipeline - from data ingestion, discovery, curation, and enrichment to analytics and augmented visual insights - and may be found in a wide range of consumer and enterprise applications across functions and domains. Some are highly visible and well-known, as in chatbots, e-commerce recommendation engines, digital twins, and AI-powered digital assistants, while others are more hidden but embedded into various workflows such as algorithmic decisions, outlier detection, exception routing, prediction machines, image recognition, or robotic process automation. One prominent example of where analytics and AI is particularly relevant is in understanding customer journeys. Understanding a customer holistically, longitudinally, and contextually provides the basis to determine what certain behavioral changes or incentives could be proposed in the future. It shows that the single view of the customer is not just a static point of view, but an analysis that mines data to determine patterns and behaviors over time. What you then have are insights into when something will happen, how it should happen, or whether there are similar individuals. If businesses are successful in creating such insights, they can leverage them to motivate up- or cross-selling or drive customer engagement to enable new value for their company.

## How do you see the “intelligence” of these solutions?

What becomes increasingly critical is that ML models get auto-tuned, which means that they use continuous feedback to learn and improve over time. This will allow us to move beyond static process automation to fundamentally enhancing service experiences. Arguably some of the most advanced businesses in this respect are the big tech companies. Auto-tuning allows them to dynamically adjust and target their offerings to provide customers with experiences they truly want to achieve. We see that most, if not all digital platform companies, leverage such ML auto-tuning capabilities since they are in possession of tremendous data assets about users. With algorithmic learning, every click, view, browse, or interaction of their customers/users across platforms serves to continuously adjust the content, the services, and the recommendations. This not only applies to e-commerce retailers, where we see it prominently but also in other contexts such as the connected home, where your thermostat is smart and learns from your past behavior or in the financial industry, where bots learn from interacting with the stock market environment to optimize trading strategies.

## Could you think of marketplaces for information objects?

We are already seeing many examples of information object marketplaces in both the enterprise and consumer space. For one, we are experiencing a proliferation of data marketplaces. Many organizations seek to augment and enrich their internal data with external data sets to create better insights. Data marketplaces match data consumers with data sellers and monetize their data by selling data and datasets directly, products/services derived from data, subscription services, or data that could be used to train AI-based products. Another example are API marketplaces, which allow providers to list their APIs and developers to discover and consume them. An exciting emerging example are algorithm marketplaces, which big tech companies, startups, and developers use to share and sell their work. GitHub, a code hosting and sharing platform acquired by Microsoft, launched a marketplace to support developers in collaborating with others and monetize their codebase. Amazon’s AWS marketplace offers models and algorithms for computer vision, speech recognition, and natural language processing. Algorithmia, recently acquired by DataRobot, built an open marketplace for ML algorithms that companies could find and insert into their applications. There are also many specialized algorithm marketplaces for specific use cases, such as Nuance AI Marketplace that offers an API for access to critical diagnostic imaging algorithms used in radiology.

## You mentioned visualization, what is included in this activity?

AI is unquestionably on everyone’s mind given the tremendous value it can unlock. At the same time, there are also some real concerns with AI, primarily related to transparency, bias, and ethics. Not understanding how and why an algorithm made a particular decision can have some serious negative impacts, as evidenced in recent well publicized examples in employment decisions, financial decisions, or medical decisions. In fact, a black box approach to data, analytics, automation, and AI can bring with it a lot of distrust, skepticism, fears, and dangers. Our global team of visualization experts, data scientists, designers, and product engineers considers a wide range of topics and application areas such as human-centered design, data experiences, immersive visual analytics, digital twins, and enterprise simulators. My position is to think of visualization not only as the last mile of analytics or as a static report, but rather as an integral interactive part across the entire end-to-end applied intelligence pipeline. Visualization is a key capability in overcoming some of these challenges. Interactive visualizations bring humans and machines together seamlessly, leveraging their respective strengths, through multi-modal interfaces, to ultimately harness the value of data, analytics, and AI when there is a human-in-the-loop. Well-designed, engineered, and interactive visualizations combined with the power of analytics and AI, augment the ability of executives, data scientists, and consumers, to make sense, discover, explore, and communicate data, to make analytics and AI explainable, to provide more trustworthy insights. Ultimately, they lead to more confident decision-making and actions. Visualizations may be used to convey complex business issues and bring strategic scenarios to life. Data scientists can leverage visualization to build better models and inspect how they are performing against desired goals. End users, such as medical professionals or supply chain managers, can rapidly and effectively consume complex contexts, such as patient charts or global supply chain risks. When shifting the focus of visualizations from purely descriptive (“what has happened or is happening”) to predictive (“what will happen”) and prescriptive (“what should be done”), we can then move from a reactionary to a more explanatory, proactive and anticipatory mode.

## Could you give us an example?

Take the example that I may be skeptical whether I will receive all the gifts I purchased for the holidays. Imagine each company had a single view of their supply chain that is not only a descriptive single view, but rather a truly dynamic, multi-level, data-driven model of their enterprise in the form of an interactive, immersive digital twin. Decision-makers would not only be able to see what is or is not happening in time, but also collaboratively explore and discuss why something is happening and decide what could be done if something occurs that questions the timely delivery. To make this come to life, you would need rich, integrated data at multiple levels and across functions and domains, combined with analytics-powered data stories that enable augmented insights to answer “what-is” and “what-if” questions. In our vision of the future control tower for the C-suite and boardrooms we aim to give decision-makers the entire spectrum for their decision-making. This includes the data and the tools, but also the complex context with numerous interdependencies in the decision space. Visualizing the underlying data and analyses is important, but even if done right, it sometimes can be simply too much to consume and lead to cognitive overload. In that case a more targeted, guided approach is needed. What if we then allowed AI tools and methods to identify and generate the most important data facts, present them in natural language form, allow decision-makers to drill-down or drill-up, and rate and like the recommendations?

## How are you approaching this in your consultancy work?

As I mentioned earlier, one of the overarching challenges for data, analytics, and AI in practice today is to move beyond the proof-of-concept and pilot stage as well as to create value for business with speed and at scale. My experiences from projects across retail, consumer goods, energy, manufacturing, finance, healthcare, and life sciences continue to point at some common elements. Specifically, they told me that the future is not just algorithmic but will also involve human-in-the-loop thinking.Although algorithms are supercritical on the automation side, we should go beyond hailing algorithms and understand where humans are necessary. This is also a profound call for ethics and responsible AI, because if we fail to explain what is going on and to uncover what is driving decision-making, the implications might be dangerous and lead to social friction. Think of insurance or credit applications that you might not receive due to some vague or even unfair algorithmic decision-making. In our work, we see that the scientific approaches to storytelling are not widespread in practice and even consulting companies require more sophistication and scale in this regard. We need to go beyond the presentation-driven dashboard-centric world of one-way communication towards a truly interactive two-way communication. Instead of broadcasting static artefacts we should be thinking of interactive documents and prototypes that bring data to life. I see much potential in applying augmented, extended, and virtual reality technologies for this purpose, but we also need to do a much better job of storytelling when building dashboards and visualizations. This poses an exciting area for collaboration with research institutions as well as innovative start-up businesses, for example in persuasive design and visual communication or computational journalism. 

## What role do you see for academia?

We are in an exciting time for people in data, analytics, and AI. We have an amazing set of opportunities to not only digitize enterprises of all sizes, functions, and industries, and geographies, but also to help them unlock that value and reshape business through a continuum of technology and experience. First, I think we are not training enough people. The growing demand for AI is outpacing the talent base that is needed. Academia needs to generate people with the right talent, skills, and experience mix that will feed this appetite. We observe that the entire industry is in hyperdrive mode and we have opportunities across all areas of AI. Educating and upskilling the future workforce and making them relevant to practice has never been more important than today. There are plenty of opportunities for I-shaped people (those that are focused in one area), T-shaped people (those that show depth in one and breadth across), and X-shaped people (those that can play a truly cross-functional role). Given that I have seen both sides of table, I am a true believer that strong industry-academic partnerships can lead to highly synergistic opportunities. We can be sure that digital transformation will evolve further over time, and this requires that we work closer with universities. I think that some of most the interesting problems and comprehensive datasets currently exist in industry: if you want data, we have data. What the research community contributes is true rigor and an innovative thinking of how to tackle fundamental problems. In industry, we often must align our speed with the pace of our clients, which means that we must deliver value for immediate needs. Academia in turn has a much longer timeframe for addressing profound questions. I would encourage most academics to find a good industry partner and I strongly advocate for professors in residence at companies. For example, we can be sure that quantum computing, the metaverse, or blockchains will have much impact on future solutions. Bringing business and academia closer together could be one of the keys to unlock the potentials of these technologies.

## What is your position regarding the big tech companies?

Because we are agnostic of the technological solutions and infrastructures and since we work with a rich partner ecosystem, my position is rather neutral. Let me distinguish between the implications for academia and for businesses. Big tech companies are unquestionably a strong driver of innovation and many if not all of them have dedicated research organizations. Since they have access to data, the problems, the infrastructure, and the talent, one of the dangers could be that these companies disrupt the traditional research and education landscape by, for instance, starting their own degree programs. What does that mean to the future of information systems, computer science or engineering programs? For universities to remain relevant, some foundational questions must be answered. For businesses, the big tech companies are often the key providers of infrastructure and solutions across the tech stack. It makes no sense for many retail, healthcare, or consumer goods companies to build their own infrastructures instead of leveraging existing offerings. The key questions, however, occur at the data and analytics level, where value is truly unlocked: who should own it, where is it hosted, and how should it be used. In our consultancy work, every digital transformation is tailored towards the need of that organization, there is no one-size fits all. We must consider existing strategies, legacy systems, and operation concepts, and determine what the to-be state should be and how to get there. Our approach typically begins with conceptualizing the foundational layer since this is the basis for creating value at scale and then we think about the activation and consumption layers in terms of data products and decision products, which could be sophisticated analytics and AI. We help our clients make informed technology decisions across the entire landscape and guide them through their digital transformation journey. One thing is clear, however, all the advice is without value if leadership is not boarded in. Even a great strategy is only as good as you are executing on it.

## What future topics would you see for research?

There are several topics that I would love to see pursued further. The real-world is quite complex and it requires a truly transdisciplinary lens. First, I would welcome to see more of a complexity lens applied to information systems research topics. We typically reduce research problems to very specific bounded aspects for many practical reasons. Yet, when we reduce the problem to something very specific, the actual underlying issues can sometimes be thought away. Complexity theory calls for thinking in terms of interdependent systems and advocates a holistic perspective on how things fit together. We already know that digital platforms and ecosystems are highly interconnected. Exploring other digital transformation topics from a complex systems perspective could lead to some novel findings and be highly valuable to practitioners. It could be the opportunity for the community to collaborate with other scientific domains as well. I think we are in a great moment for complex systems as Giorgio Parisi was awarded the 2021 Nobel prize in physics for his work in this field. Second, real-world AI problems are truly cross-functional and multi-disciplinary. They are not only the domain of data scientists or ML engineers, but also need designers, product engineers, strategists, operations researchers, human-computer scientists, ethicists, policymakers, and change management specialists. Value is not just created within an organization, but in collaboration with an open ecosystem of stakeholders. Future information systems research should explore the multi-dimensionality of these emerging enterprises and help discern how they work, why they work, and under what conditions they fail. Lastly, designing effective, engaging, and aesthetic human-data interfaces will continue to be important in the era of AI. What role can these analytics and AI-powered interfaces play in topics such as AI responsibility, sustainability, misinformation, or the future of climate, agriculture, biotech, and healthcare? If you think about the energy requirements that are needed for AI, how much computer power should be used for every ML model and how much CO2 is it worth wasting? Each of these questions not only require a technological lens, but also a truly social, political, and economic lens. The opportunity here for IS research is to create a bridge across different disciplines.

Dear Rahul, thank you for the interview!
